# The effects of a formal exercise training programme on salivary hormone concentrations and body composition in previously sedentary aging men

**DOI:** 10.1186/2193-1801-2-18

**Published:** 2013-01-22

**Authors:** Lawrence D Hayes, Fergal M Grace, Nick Sculthorpe, Peter Herbert, John WT Ratcliffe, Liam P Kilduff, Julien S Baker

**Affiliations:** 1 Institute of Clinical Exercise and Health Science, University of the West of Scotland, Hamilton, Scotland; 2 School of Human Sciences, London Metropolitan University, 166-220 Holloway Road, London, N7 8DB UK; 3 Department of Sport and Exercise Sciences, University of Bedfordshire, Bedfordshire, UK; 4 School of Sport, Health and Outdoor Education, Trinity Saint David, University of Wales, Wales, UK; 5 Department of Sports Science, Swansea University, Swansea, UK

**Keywords:** Cortisol, Testosterone, Sarcopenia, Aging, Physical activity

## Abstract

Alteration in body composition, physical function, and substrate metabolism occur with advancing age. These changes may be attenuated by exercise. This study examined whether twenty eight, previously sedentary males (62.5 ± 5.3 years of age; body mass of 89.7 ± 16.4 kg) adhering to the ACSM minimum guidelines for aerobic exercise for six weeks would improve exercise capabilities, body composition and salivary hormone profiles. After six weeks of adhering to the guidelines, salivary testosterone and vo_2max_ (absolute and relative) increased (p < 0.05), whilst body fat percentage and body mass decreased (p < 0.05). Peak power output, fat free mass and cortisol values were not significantly different. Interestingly, salivary testosterone correlated inversely with body fat percentage (R^2^ = .285, p = 0.011). These results suggest that despite previous inactivity, older males can achieve improvements in cardiorespiratory fitness, body composition and anabolism by adhering to simple lifestyle changes.

## Introduction

Advancing age is associated with alterations in body composition, physical function, and substrate metabolism. Loss of skeletal muscle mass (sarcopenia) contributes to declines in muscle strength and function along with diminished quality of life (Sattler et al., 2009). Age-related body composition changes and losses in muscle mass play important roles in frailty (Wu et al., 2010).

Coincident with the age-related deteriorations in clinical status, endogenous production of anabolic hormones declines (Harman et al., 2001). Testosterone plays key roles in regulating muscle mass and fat free mass (Hayes et al., 2010), and it declines with age in both men (Feldman et al., 2002) and women (Davison et al., 2005). Harman et al. (2001) reported that total and free testosterone decreased linearly with age from 21-45 yr onwards and approximately 25-30% of men over 60 yr age have hypogonadal testosterone levels. Testosterone insufficiency has been shown to lead to increased visceral fat mass, decreased lean body mass, and decreased muscle strength (Wu et al., 2010). It has been widely reported that testosterone replacement increases synthesis of myofibrillar proteins, total body cell mass, muscle strength (Urban et al., 1995), and reduced trunk and visceral fat, blood pressure, lipids, and improves insulin sensitivity (Brand et al., 2011). Increasing observational evidence also suggests a protective role of endogenous testosterone in men’s cardiovascular health (Brand et al., 2011). Some research has shown that circulating concentrations of anabolic hormones such as testosterone in older men are related to increased levels of physical activity, muscle function, and aerobic power (Herbst and Bhasin, 2004). Therefore, to counter the age-associated decline in anabolic hormones, regular exercise has been suggested as a possible nonpharmacological intervention to elevate the concentration of circulating anabolic hormones in older individuals.

Exercise has been observed to improve quality of life by decreasing body fat and obesity, increasing muscle strength, improving balance, gait, and mobility, decreasing the likelihood of falling, improving psychological health and reducing arthritis pain. Exercise has also been implicated in reducing the risk of developing coronary heart disease, hypertension, osteoporosis, cancer, and diabetes (Wroblewski et al., 2011). The ACSM guideline for aerobic exercise in older individuals is “a minimum of 5 d∙wk^-1^ of moderate intensity, or a minimum of 3 d∙wk^-1^ of vigorous intensity exercise, and to accumulate at least 30 min∙d^-1^ of moderate-intensity activity, in bouts of at least 10 min each; continuous vigorous activity for at least 20 min∙d^-1^” (Nelson et al., 2007). It is apparent that resistance exercise can maintain muscle mass and strength in older individuals. It has also been shown that testosterone supplementation can increase muscle mass in the elderly. As exercise is known to increase levels of testosterone (Caruso et al., 2012; Lovell et al., 2012), the primary aim of the present investigation was to examine whether exercise could produce improvements in common symptoms of the aging process. Participants adhered to the ACSM guideline for aerobic exercise in older individuals for six weeks. A further aim was to investigate whether salivary hormones may reflect physiological changes associated with fitness.

## Materials and methods

Participants were tested at two points, separated by six weeks. In the six weeks between testing sessions, participants were prescribed with a combination of aerobic, anaerobic and resistance training activities. Participants were instructed to exercise twice a week for two weeks and by the third week achieve 130 min∙wk^-1^ of moderate exercise. For the final three weeks, ∼150 min∙wk^-1^ of moderate to vigorous work was reported. Exercise activities were overseen by a member of the research team. To ensure levels of low, moderate and some high intensity exercise, intensity was confirmed using participant heart rates (Polar, Finland).

All participants met the ACSM minimum guidelines for aerobic exercise but did not excessively exceed them. Activities consisted of walking, cycling, hill walking and resistance training. Participants achieved an average heart rate (HR) between 70 and 80% of HR_max_ by the end of the programme (Table 1). RPE's were also noted at the end of the sessions. These ranged between 13 and 15 during the final 3 weeks; somewhat hard to hard.

**Table 1:** **Mean ± SD HR expressed as a percentage of HR**_**max**_**during the six weeks of exercise. No significant differences were observed**

Week 1	Week 2	Week 3	Week 4	Week 5	Week 6
74.7 ± 4.2	75.6 ± 3.9	75.4 ± 4.5	74.5 ± 4.7	74.0 ± 5.0	75.0 ± 5.4

On both testing days, participants arrived at the same time of day to exclude diurnal variation of measurements (Hayes et al., 2010). Prior to experimental data collection participants were fully habituated and familiarized with data collection and testing procedures. This was done to eliminate any learning effect. The order of measurements were; saliva sampling, body composition, peak power assessment, and VO_2max_ assessment. A minimum of 5 min recovery was allowed between the peak power and VO_2max_ assessment to allow replenishment of the ATP/PCr system. The investigation met the ethical standards as outlined previously (Harriss and Atkinson, 2011) and was approved by the University of the West of Scotland Ethics Committee.

### Participants

Twenty eight males 62.5 ± 5.3 years of age, with a stature of 174.5 ± 6.4 cm, and body mass of 89.7 ± 16.4 kg participated. All participants were sedentary (they were not part of an organized exercise program and did not meet the ACSM minimum guidelines for aerobic exercise). Participants had abstained from alcohol, caffeine and exercise for 24 h prior to the investigation. Participants woke up at ~07:30 and arrived at the laboratory in a four-hour fasted condition as requested according to self-reporting. A conventional overnight fast was used unless participants were scheduled to arrive in the afternoon, in which case a four hour fast was used. To increase reliability, participants were allocated the same appointment time on both visits to the laboratory. Participants reported to the laboratory on two separate days, separated by six weeks. Preceding data collection and exercise, all subjects had an ECG following ACSM guidelines, and all completed health history questionnaires. All subjects were deemed fit to participate in the study, and were signed of as healthy by a medical Doctor.

### Body composition

Height was measured to the nearest 0.1 cm using a stadiometer (Seca, Birmingham, UK) and body mass to the nearest 0.1 kg (Tanita MC-180MA Body Composition Analyzer, Tanita UK Ltd.). A multi frequency bioelectrical impedance analyzer ((BIA) Tanita MC-180MA Body Composition Analyzer, Tanita UK Ltd.) was used to measure body composition. Coefficient of variance (CV) of the impedance measure was 0.4%. The Tanita GMON software (v1.7.0) generated values for fat free mass (FFM) and body fat percentage. Individual haematocrit (Hct) concentrations were used as indirect measures of hydration status to validate BIA results. Concentrations were 44.8 ± 4.1 and 46.0 ± 2.1 for week 0 and week 6 respectively. Values obtained from BIA were supported by skinfold measurements using Harpenden calipers.

### Saliva collection and analysis

Methods have been described previously (Hayes et al., 2012). Briefly, whole salivary samples of approximately 1.8 mL were collected via expectoration into graduated 2 ml cryovials (Salimetrics, State College, PA). To prevent potential blood contamination of saliva, resulting in an overestimation of hormone concentrations, participants were advised to avoid brushing their teeth and drinking hot fluids 2 h prior to reporting to the study venue. Salivary samples were collected and transported to a freezer immediately where they remained at -80ºC until assay. Samples were assayed in duplicate (without separation or extraction) for cortisol and testosterone using commercially available immunoassay protocols (Salimetrics, State College, PA). All samples were assayed for salivary cortisol using a high-sensitivity enzyme immunoassay (EIA) (Salimetrics, State College, PA) with a lower limit of sensitivity of <0.19 nmol/L, and average intra- and inter-assay coefficients of variation 4.13% and 8.89%, respectively. Saliva samples were assayed for testosterone using EIA with a lower limit of sensitivity of 5.20 pmol/L, and average intra- and inter-assay coefficients of variation less than 10% and 15%, respectively.

### Peak power assessment

An adaption of the Wingate anaerobic test consisted of a 10-s maximal sprint against constant resistance on an air-braked cycle ergometer (Wattbike Ltd., Nottingham, UK). For each subject the damper resistance was set at 10 (described as “heavy gearing” by the manufacturer). Considering the participants were mostly aged 60-70 yr old, it would have been unlikely that any would have achieved over 1350 W, which was the case. All subjects reached their peak power by 7 s, usually by 5 s. All subjects completed a standardized 3-min warm-up involving pedalling at 60 rpm interspersed with three 2- to 3-s all-out sprints. A recovery period of 5 min was allowed between the warm-up and the test. The test commenced from a standing start. Subjects were verbally encouraged throughout the test to avoid pacing and to sustain their supramaximal effort throughout the test. The power output was calculated each second for the duration of the test and peak power over 1 s was recorded.

### Vo_2max_ assessment

A 3 min warm up on an air-braked cycle ergometer (Wattbike Ltd., Nottingham, UK), preceded the ramped protocol. Participants commenced their VO_2max_ test on different cadences dependent on their power output values and maintained these cadences throughout the test until exhaustion. The cadences were 70, 75, 80, or 85 rpm and work-rate was increased every minute by raising the damper setting by one until the participant was unable to continue despite vigorous encouragement. The participants cycled at a self-selected pedal rate which remained constant throughout the test (70–90 rpm). Pulmonary gas exchange was measured breath-by-breath throughout the exercise test. Participants breathed through a low-resistance volume transducer, which had a dead space of 90 ml. Gas was continuously drawn down a capillary line into rapid-response gas analysers (Metamax II, Cortex, Leipzig, Germany) and gas exchange variables were calculated and displayed breath-by-breath after accounting for the delay between the volume and concentration signals. The volume transducer was calibrated before each test with a 3-l calibration syringe and the analysers were calibrated with precision-analysed gases that spanned the expected range of expired O_2_ and CO_2_ concentrations. HR was recorded every 5 s using short-range telemetry. A fingertip blood sample was collected into a portable automated lactate analyser (Lactate Pro, Arkray, Inc., Kyoto, Japan) within 45 s of the termination of exercise. VO_2max_ was calculated as the highest 20 s value achieved during the test. The ramp test data for each individual participant were subsequently examined to assess the extent to which the secondary criteria (based on maximum values for RER, HR and blood [lactate]) could be used to confirm, or otherwise, the measured VO_2max_: For RER, the VO_2_ values corresponding to RER values of 1.10 and 1.15 were calculated and compared to the VO_2max_: For HR, the number and proportion of participants who satisfied the criterion of achieving a HR which was within ±10 b/min (or 5%) of the age-predicted maximum (220-age in years) was calculated. Finally, the number and proportion of participants who satisfied the criterion of achieving a peak blood [lactate] of >6 mM was established.

### Statistical analysis

Data were analyzed using SPSS (version 18) (IBM North America, New York, NY, USA). Conventional methods, mean, standard deviation (SD), repeated measures analysis of variance (ANOVA) with bonferroni correction, and Pearson’s correlation were used to analyze participant descriptive and experimental data. Hormonal and exercise-test performance data were analyzed with ANOVA. Significance was set *a priori* at p ≤ 0.05.

## Results

Aerobic capacity increased (p < 0.05) after six weeks activity compared to baseline (Table 2). Peak power output was not significantly different. There were significant increases in resting salivary testosterone (p < 0.05) from baseline to six weeks but not cortisol. Percentage body fat decreased (p < 0.05), as did body mass (p < 0.05). However, FFM was not significantly altered. Salivary testosterone correlated inversely with body fat percentage (*R*^*2*^*= .285, p = 0.011*). Vo_2max_ correlated inversely with FFM *(R*^*2*^ = .*293, p = 0.007*), total body mass (*R*^*2*^*= .551, p < 0.001*), and body fat percentage (*R*^*2*^*= .711, p < 0.001*); yet positively with testosterone (*R*^*2*^*= .268, p = 0.017*) and peak power output (*R*^*2*^ = .28*1, p = 0.010*).

**Table 2:** **Exercise performance, hormonal, and body composition values after six weeks of the ACSM minimum guidelines for aerobic exercise**

Variable	Week 0	Week 6
VO_2max_ (ml·kg·min^-1^)	26.85 ± 5.31	28.55 ± 4.80
		*p = .012*
VO_2max_ (L·min^-1^)	2.38 ± 0.46	2.51 ± 0.43
		*p = .035*
Peak power output (W·kg^-1^)	7.58 ± 1.68	7.77 ± 1.58
		*p = .580*
Peak power output (W)	672.80 ± 157.98	682.7500 ± 152.13
		*p = .344*
Testosterone (pmol·L^-1^)	378.80 ± 127.33	484.30 ± 182.35
		*p = .010*
Cortisol (nmol·L^-1^)	2.76 ± 1.47	3.20 ± 1.45
		*p = .156*
Total body mass (kg)	90.44 ± 18.09	89.28 ± 17.36
		*p = .003*
FFM (kg)	63.76 ± 8.69	64.70 ± 8.01
		*p =.084*
Body fat percentage (%)	28.73 ± 5.00	26.69 ± 5.20
		*p =.003*

Values are mean ± SD; VO_2max_ = maximal oxygen uptake, FFM = fat free mass. *p values are reported for differences between week 0 and week 6.*

## Discussion

Our data suggest that six weeks of increased physical activity in previously sedentary men can improve body composition, exercise performance and testosterone levels. As suggested by Herbst and Bhasin (2004), testosterone was related to increased levels of physical activity and aerobic power in older men. Contradictory to the present investigation, Lovell et al. (2012) reported that 16 weeks of resistance training or 16 weeks of aerobic training at 70% of VO_2max_ for 45 min three times per week did not provide sufficient stimulus to significantly increase resting testosterone or free testosterone in older men. Methodological discrepancies may explain these different findings. Firstly, Lovell et al. (2012) recruited participants who were moderately active with walking and gardening as their main activities whereas the present investigation recruited participants who self-reported no organised physical activity throughout a week. However, the most reasonable explanation is the age difference of participants. In the current investigation, we recruited participants aged 55-74 years to participate, whereas Lovell et al. (2012) tested participants 70 – 80 years. As testosterone is known to decline with increasing age (for a review see Wroblen 1991), at approximately 1% per year after the age of 40 years (Gray et al., 1991), the ability of increased exercise to elevate testosterone may be blunted in older individuals.

As salivary testosterone reflects the unbound (free), bioavailable steroid hormone fraction found in the general blood circulation, the increase found in the present study has important implications for older individuals. The higher free testosterone, may indicate an improved “bioactivity status” in the blood of the participants. Higher free testosterone levels have been associated with greater bone mineral density in men (Murphy et al., 1993) and are reportedly inversely associated with fat mass (Abbasi et al., 1998). Free testosterone concentration is also a significant independent predictor of lean body mass and muscle strength (Baumgartner et al., 1999). Salivary testosterone increases found in the present study, coupled with no observed change in resting cortisol levels suggest an enhanced anabolic environment in participants, however no increase in FFM was noted. Nevertheless, reductions in total body mass and body fat percentage were observed. The relationship between testosterone and body fat percentage observed in the current investigation is in agreement with the findings of Abbasi et al. (1998) who noted that total testosterone and free testosterone have been identified as predictors of FFM and total adipose mass.

Participants experienced increases in VO_2max_ after the six weeks of increased activity. This was not an unexpected result as previous studies have found that even moderate exercise can increase VO_2max_ of older men (Niederseer et al., 2011). As absolute values were increased from baseline, we can conclude that cardiorespiratory adaptations did occur rather than relative values increasing purely due to body mass decreases. Peak power output (relative and absolute) remained unaltered after six weeks of increased activity. However, this is unsurprising as participants did not engage in high intensity activities likely to improve power output. As with FFM, there was a trend for increased peak power output after the six week intervention but this did not reach significance.

Despite the encouraging findings of the present investigation, a limitation was the relatively small sample size and short observation period. Therefore, future investigations utilizing a longer study period with a larger sample size may be beneficial.

In conclusion, our data demonstrates that six weeks of the ACSM minimum guidelines for aerobic exercise increase resting salivary testosterone of previously inactive older men. Furthermore, body composition and maximum oxygen uptake improvements were noted after moderate lifestyle changes. As sports medicine clinicians, we must encourage people to become or remain active at all ages. It is crucial that increased activity levels are maintained habitually as improvements in VO_2max_ are lost once the stimulus of exercise is removed (Lovell et al., 2012). This study and those reviewed here, document the possibility to improve physical fitness across the ages via simple lifestyle changes.
